# Supplementary material for: Dual role of glycosylation in resistance to CD4-binding site broadly neutralizing antibodies
Source: J Virol. 2026 Apr 3;100(5):e02093-25. doi: 10.1128/jvi.02093-25 (PMC13185609; doi:10.1128/jvi.02093-25)
Supplement: Figures S1 and S2 — Figure 1: Effect of N276 glycan removal on neutralization profiles of bNAb panel. Figure 2: Effect of producer cell on mutant neutralization sensitivity. [file jvi.02093-25-s0001.docx]

**Supplementary Figure 1. Effect of N276 glycan removal on neutralization profiles of bNAb panel.** A. Heat map of the IC50 of each virus-antibody pairing for the wildtype virus compared to N276D mutant virus. B. non-CD4bs bNAb IC50 change in the global panel of HIV env reference strains when N276D mutation is inserted. Graphs are a visualization of the values in the heatmap.

**Supplementary Figure 2. Effect of producer cell on mutant neutralization sensitivity.** Heat map of the IC50 of TRO.11 and 246.F3 with each antibody pairing for the wildtype virus compared to N276D and N276K mutant viruses. Values are means from two independent experiments. Gray indicates neutralization assays not performed.
